# A Maternal High-Fat Diet Induces DNA Methylation Changes That Contribute to Glucose Intolerance in Offspring

**DOI:** 10.3389/fendo.2019.00871

**Published:** 2019-12-13

**Authors:** Qian Zhang, Xinhua Xiao, Jia Zheng, Ming Li, Miao Yu, Fan Ping, Tong Wang, Xiaojing Wang

**Affiliations:** Key Laboratory of Endocrinology, Ministry of Health, Department of Endocrinology, Peking Union Medical College, Peking Union Medical College Hospital, Chinese Academy of Medical Sciences, Beijing, China

**Keywords:** DNA methylation, insulin receptor substrate, MAPK, maternal high fat diet, epigenetics

## Abstract

**Scope:** Overnutrition *in utero* is a critical contributor to the susceptibility of diabetes by programming, although the exact mechanism is not clear. In this paper, we aimed to study the long-term effect of a maternal high-fat (HF) diet on offspring through epigenetic modifications.

**Procedures:** Five-week-old female C57BL6/J mice were fed a HF diet or control diet for 4 weeks before mating and throughout gestation and lactation. At postnatal week 3, pups continued to consume a HF or switched to a control diet for 5 weeks, resulting in four groups of offspring differing by their maternal and postweaning diets.

**Results:** The maternal HF diet combined with the offspring HF diet caused hyperglycemia and insulin resistance in male pups. Even after changing to the control diet, male pups exposed to the maternal HF diet still exhibited hyperglycemia and glucose intolerance. The livers of pups exposed to a maternal HF diet had a hypermethylated insulin receptor substrate 2 (*Irs2*) gene and a hypomethylated mitogen-activated protein kinase kinase 4 (*Map2k4*) gene. Correspondingly, the expression of the *Irs2* gene decreased and that of *Map2k4* increased in pups exposed to a maternal HF diet.

**Conclusion:** Maternal overnutrition programs long-term epigenetic modifications, namely, *Irs2* and *Map2k4* gene methylation in the offspring liver, which in turn predisposes the offspring to diabetes later in life.

## Introduction

The incidence of type 2 diabetes mellitus (T2DM) is dramatically increasing. T2DM has become a major public health problem worldwide. It is clear that genetic factors play important roles in the incidence of T2DM. However, the genetic loci identified by genome-wide association studies (GWASs) can account for only a small proportion of T2DM (<10%) (1, 2). Heritable factors cannot explain the dramatic increase in T2DM, thus recent research has focused on lifestyle as a major factor for the incidence of T2DM (3).

Among lifestyle factors, prenatal and postnatal nutrition imbalances lead to epigenetic programming, which is associated with increased T2DM incidence, including both undernutrition and overnutrition (4). Maternal nutrition is important in determining susceptibility to metabolic disease (5). Epidemiological studies in humans revealed that both under- and overnutrition of the mothers during pregnancy will have far-reaching and long-term outcomes for the health of the offspring in adult life (6, 7). For example, glucose intolerance and insulin resistance were reported in offspring whose mothers were exposed to undernutrition during gestation in the well-known study of the Dutch famine, demonstrating an association (8). Similarly, maternal obesity is associated with increased susceptibility to T2DM in offspring (9, 10). Female rat pups from mothers with high-fat (HF) diet-induced obesity were more prone to obesity (11). We have reported that a maternal low-chromium diet can also program the development of diabetes in offspring (12).

Intrauterine programming has been proposed in which the maternal nutrition environment could affect the metabolism of the offspring throughout life (13). The prenatal and early postnatal period is considered a critical time for adult life. Moreover, the maternal nutrition environment and health status induce epigenetic modifications that affect the incidence of T2DM in offspring. The Developmental Origins of Health and Disease (DOHaD) concept has been proposed, and epigenetic mechanisms are considered to possibly underlie DOHaD (14, 15). Development programming in the early life period can increase the risk of T2DM in later life (14). Epigenetic modifications mainly consist of DNA methylation, histone modifications and non-coding RNAs. These epigenetic modifications can affect gene expression via environmental changes (16). DNA methylation is defined as the addition of a methyl group to a cytosine, usually in CpG islands (17). DNA methylation at CpG-rich promoters or gene regulatory regions is normally associated with the inhibition of gene expression (17).

Recently, a small number of studies have described the role of DNA methylation changes in fetal programming to metabolic disease in human and animal models. In human research, newborns with obese parents had altered methylation in multiple imprinted genes in their cord blood (18). In animal research, the results were not consistent. In one study, exposure to a maternal HF diet caused DNA hypermethylation in offspring mouse livers (19). However, Cannon et al. did not detect any effect of the maternal diet on DNA methylation in the male mouse liver (20). Another group reported that exposure of offspring to a maternal HF diet could remodel the hepatic epigenome, if they changed to a control diet during the postweaning period (21). Hence, the precise molecular mechanisms underlying the epigenetic changes induced by a maternal HF diet have not yet been thoroughly identified.

Thus, in this study, we sought to determine whether genome-wide changes in DNA methylation occur in the livers of offspring exposed to a maternal HF diet and a postweaning control diet. The liver was chosen because it is essential for maintaining metabolic homeostasis (22). We performed a genome methylation array to identify differentially methylated genes in 8-week-old postweaning control diet-fed offspring from HF diet-fed or control diet-fed dams. Differentially methylated genes were assessed in the offspring mouse genome to determine the epigenetic mechanism responsible for the maternal HF diet effects.

## Materials and Methods

### Animal Grouping and Treatments

All research procedures involving animals were approved by the Animal Care Committee of Peking Union Medical Hospital (Permit Number: MC-07-6004). Male and virgin female C57BL6/J mice were purchased from the Institute of Laboratory Animal Science, Chinese Academy of Medical Sciences and Peking Union Medical College (Beijing, China). All animals were housed under specific pathogen-free conditions. Mice were kept in a controlled environment (25 ± 1°C) under a 12-h light/dark cycle and allowed food and water *ad libitum*.

**Figure 1:**
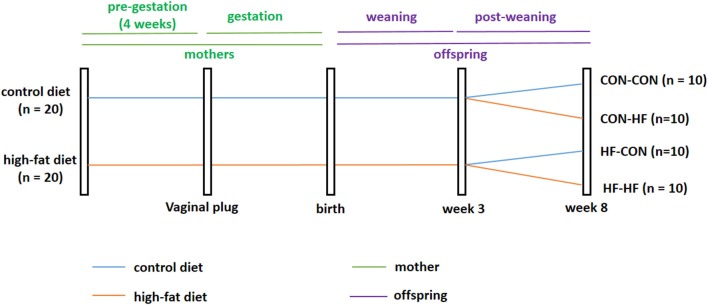
Timeline of animal experiment. CON-CON: control diet mother-post-weaning control diet; CON-HF, control diet mother-post-weaning high-fat diet; HF-CON, high-fat diet mother-post-weaning control diet; HF-HF, high-fat diet mother-post-weaning high-fat diet.

Five-week-old virgin females (*n* = 40) were divided into two groups at random. One group of mice was fed a standard AIN93G control diet (CON group, *n* = 20, Research Diets, Inc.; 16, 64, and 20% of calories from fat, carbohydrate, and protein, respectively), while the other group was fed a HF diet (*n* = 20; Research Diets, Inc.; 45, 35, and 20% of calories from fat, carbohydrate, and protein, respectively). Male mice were fed a normal diet throughout the experiment. After 4 weeks, female mice were housed overnight with males of the same age to mate at a ratio of 2:1 in each cage. The presence of a vaginal plug the following morning indicated the first day of pregnancy. During gestation and lactation, the diet scheme did not change. On postnatal day 21, one male pup was selected randomly from each dam. Male pups from control diet-fed dams were weaned onto the control diet (CON-CON, *n* = 10) or HF diet (CON-HF, *n* = 10). Meanwhile, male pups from HF diet-fed dams were weaned onto the control diet (HF-CON, *n* = 10) or HF diet (HF-HF, *n* = 10). This process created four groups of pups: CON-CON group, CON-HF group, HF-CON group, and HF-HF group. All animals were sacrificed at 8 weeks of age, and the livers were immediately collected and snap frozen in liquid nitrogen and then stored at −80°C. The animal experiment timeline is shown in Figure 1.

### Body Weight, Fasting Blood Glucose, Oral Glucose Tolerance Test (OGTT), and Insulin Analysis

Body weight was measured at weaning time in mothers and 8 weeks of age in pups. Fasting blood glucose (Contour TS glucometer, Bayer, Hamburg, Germany) and plasma insulin (ELISA, Millipore, Billerica, MA) levels were measured at 8 weeks of age. Insulin sensitivity was assessed using the HOMA-IR as previously described (23). After 10 h of food deprivation, the 8-week-old offspring underwent OGTT, and the blood glucose concentrations were immediately measured with a glucometer at 0, 30, 60, and 120 min post gavage (2.0 g/kg). The area under the glucose tolerance curve (AUC) of the OGTT was calculated as previously described (23).

### DNA Methylation Profiling Using Array

To determine the effect of maternal HF diet on DNA methylation in offspring livers, genomic DNA was extracted from the livers of HF-CON and CON-CON pups (*n* = 3 in each group, selected randomly from different dams) using a DNeasy Blood & Tissue Kit (Qiagen, Fremont, CA). Samples of genomic DNA were sonicated into random fragments in a size range of ~100–500 bp. Immunoprecipitation of methylated DNA fragments (MeDIP) was performed using a mouse monoclonal anti-5-methylcytosine antibody (Diagenode). The total input and immunoprecipitated DNA were labeled with Cy3- and Cy5-labeled random 9-mers, respectively, and hybridized to an Arraystar Mouse ReqSeq Promoter Array (Arrarystar Inc., Rockville, MD), which contains 22,327 well-characterized RefSeq promoter regions [from ~-1,300 to +500 bp of the transcription start sites (TSSs)] totally covered by ~180,000 probes. Scanning was performed with an Agilent Scanner G2505C (Agilent Technologies, Waldbronn, Germany).

### Methylation Enrichment and Peak-Finding

The results were obtained using a sliding-window (750 bp) peak-finding algorithm provided by NimbleScan v2.5 (Roche NimbleGen). NimbleScan detects peaks by searching for at least two probes above a minimum cutoff *p*-value (–log10) of 2. Peaks within 500 bp of each other are merged. The M’ value was calculated for each probe to compare differentially enriched regions between the HF-CON and CON-CON groups as follows:

M’ = Average(${\text{log}}_{2}^{\text{MeDIP}\left(\text{HF}-\text{CON}\right)/\text{Input}\left(\text{HF}-\text{CON}\right)}$)−Average(${\text{log}}_{2}^{\text{MeDIP}\left(\text{CON}-\text{CON}\right)/\text{Input}\left(\text{CON}-\text{CON}\right)}$).

The differential enrichment peaks (DEPs) called by the NimbleScan algorithm were filtered according to the following criteria:

1. At least one of the two groups has a median (log_2_ MeDIP/Input) ≥0.3 and M’ > 0.
2. At least half of the probes in a peak may have a coefficient of variability (CV) ≤ 0.8 in both groups.

To separate strong CpG islands from weak CpG islands, promoters were categorized into three levels: high CpG promoters/regions (HCPs), intermediate CpG promoters/regions (ICPs), and low CpG promoters/regions (LCPs) (24).

### Pathway Analysis

DAVID Bioinformatics Resources 6.7 [http://david.abcc.ncifcrf.gov/ (25)] was used to perform Kyoto Encyclopedia of Genes and Genomes (KEGG) pathway and Gene Ontology (GO) functional enrichment analyses for the differentially methylated genes (DMGs).

### Bisulfite Sequencing PCR (BSP)

For validation of the methylation array, bisulfite conversion of genomic DNA from offspring livers in the four groups (*n* = 10 in each group) was conducted using a kit (Zymo Research, CA). The primers were designed using Methyl Primers Express software 1.0 (Applied Biosystems, Foster City, CA) and are shown in Table 1. The resulting PCR products were purified using a QIAquick Gel Extraction Kit (Qiagen) and cloned into the pMD18-T vector (Takara, Shiga, Japan). Individual clones were grown, and plasmids were purified using a PureLink Miniprep Kit (Invitrogen, Thermo Scientific Inc., Waltham, MA). At least 10 clones from each mouse were selected and sequenced.

**Table 1:** PCR primer for bisulfite sequencing.

**Gene**	**Accession number**	**Primer sequences (from 5^**′**^ to 3^**′**^)**	**Production size**	**CpG number**
*Irs2*	NM_001081212	F: 5′-TTTAAGGGTATTTTTGGTTTGG−3′ R: 5′-ACCATTCACTTATCAAATTCCC−3′	301	30
*Map2k4*	NM_001316367	F: 5′-TGTTTTTTGATTTTTTTTTTGG−3′ R: 5′-AAAAACTTACAACCCCAAAACT−3′	439	7

*Irs2, insulin receptor substrate 2; Map2k4, mitogen-activated protein kinase kinase 4*.

### Quantitative Reverse-Transcription PCR

Total RNA from offspring livers in the four groups (*n* = 10 in each group) was isolated using a Qiagen RNeasy Mini Kit (Qiagen, Germantown, MD) according to the manufacturer's instructions. Reverse transcription was conducted with total RNA using the TaKaRa RT kit (TaKaRa, Shiga, Japan). Real-time PCR was performed on an ABI 7900 Real-Time PCR Detection System (Applied Biosystems, Foster City, CA) using the comparative Ct method (2^−ΔΔCt^). The relative mRNA expression levels of target genes were normalized to *Gapdh*. The sequences of the primers used are shown in Table 2.

**Table 2:** qPCR primer.

**Gene**	**Accession number**	**Primer sequences (from 5^**′**^ to 3^**′**^)**	**Production size**
*Irs2*	NM_001081212	F: 5′- CGAGTCAATAGCGGAGACCC−3′ R: 5′ CCCCTGAGACCCTACGGTAA−3′	119
*Map2k4*	NM_001316367	F: 5′- TCTGTGAAAAGGCACAAAGTAAGC−3′ R: 5′- TCTCAGTCTCTCTATGTGTGGGT−3′	134

*Irs2, insulin receptor substrate 2; Map2k4, mitogen-activated protein kinase kinase 4*.

### Statistical Analysis

The results were statistically analyzed by Prism 5.0 (GraphPad Software Inc., San Diego, CA). All values are presented as the mean ± SEM. Dam body weights were compared by Student's *t* test. Body weight, blood glucose, plasma insulin, HOMA-IR, methylation, and mRNA expression in offspring were analyzed by two-way ANOVA (maternal diet x offspring diet). Statistical significance was defined as *P* < 0.05.

## Results

### HF Dams had Greater Body Weight

Dams in the HF group had higher body weights than those in the CON group at weaning time (23.33 ± 1.40 g vs. 19.17 ± 1.14 g, *P* < 0.01).

### Maternal HF Diet Did Not Affect Body Weight in Male Mice

**Figure 2:**
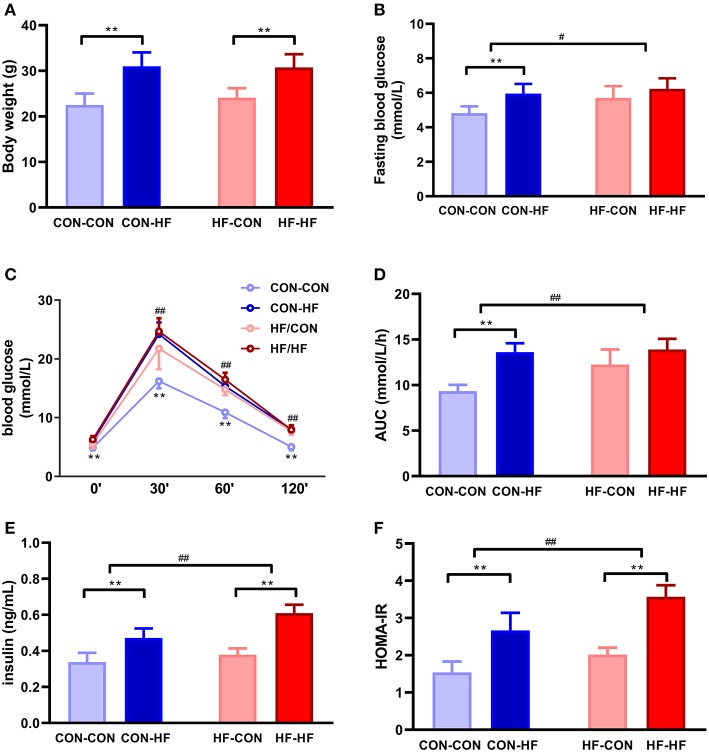
The effect of maternal high-fat diet on metabolic variables of male mice offspring. **(A)** body weight at weaning; **(B)** fasting blood glucose; **(C)** oral glucose tolerance test (OGTT); **(D)** area under curve (AUC) in OGTT; **(E)** plasma insulin; **(F)** HOMA-IR. **P < 0.01 offspring diet effect; ^#^*P* < 0.05; ^*##*^*P* < 0.01 maternal diet effect. Values are mean ± SEM (*n* = 10). CON-CON: control diet mother-post-weaning control diet; CON-HF, control diet mother-post-weaning high-fat diet; HF-CON, high-fat diet mother-post-weaning control diet; HF-HF, high-fat diet mother-post-weaning high-fat diet.

At 8 weeks of age, the mean body weight of mice in the CON-HF and HF-HF groups was significantly higher than that of CON-CON and HF-CON mice (*P* < 0.01, Figure 2A). There was no significant maternal HF diet effect on offspring body weight (*P* > 0.05, Figure 2A).

### Male Mice From HF Dams Exhibited Glucose Intolerance and Insulin Resistance

Fasting blood glucose was at similar higher levels in all HF groups (*P* < 0.01, Figure 2B). Offspring from HF diet-fed dams displayed higher fasting blood glucose (*P* < 0.05, Figure 2B). The HF diet caused significant glucose intolerance in offspring from control diet-fed dams (*P* < 0.01, Figure 2C). In particular, offspring from HF diet-fed dams had higher blood glucose levels at 30, 60, and 120 min and greater AUCs during OGTTs (*P* < 0.01, Figures 2C,D). Even when they were fed with a control diet, offspring from HF diet-fed dams had higher blood glucose levels and AUCs (*P* < 0.01, Figures 2C,D). The postweaning HF diet interacted with the maternal HF diet to increase fasting plasma insulin levels and HOMA-IR (*P* < 0.01, Figures 2E,F).

### Intrauterine Environment of HF Damsaffects DNA Methylation Patterns in Male Mice

**Figure 3:**
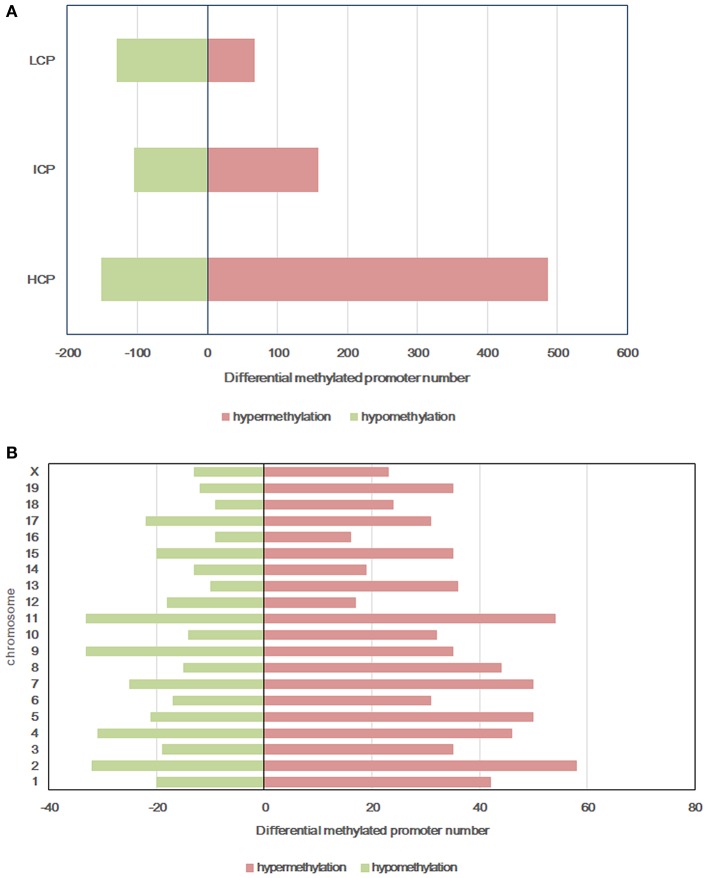
Differentially methylated promoters between HF-CON group and CON-CON group. **(A)** CpG density of differentially methylated promoters. **(B)** Chromosomal distribution of differentially methylated promoters. Red: differentially hypermethylated promoters; Green, differentially hypomethylated promoters. Classification of all promoters with high (HCP), intermediated (ICP), and low (LCP) CpG content.

All microarray data have been deposited into the gene expression omnibus (GEO ID: GSE136814). To explore the mechanism of glucose intolerance and insulin resistance observed in the offspring from dams fed a HF diet, we performed methylation arrays on the livers of the HF-CON and CON-CON groups (*n* = 3). We found that a total of 1,099 differentially methylated regions (DMRs, 955 annotated genes) were identified on 20 chromosomes in the HF-CON group compared with the CON-CON group. Among these DMRs, 713 were hypermethylated and 386 were hypomethylated. Among the hypermethylated promoters, 487 (68.33%) were located in HCPs, 158 (22.1%) in ICPs, and 68 (9.5%) in LCPs. Among the hypomethylated promoters, 151 (39.1%) were located in HCPs, 105 (27.2%) in ICPs, and 130 (33.7%) in LCPs (Figure 3A). DMRs were mainly located on chromosomes 2, 4, 7, 9, and 11 (Figure 3B).

### DMR-Related Gene Analysis

**Figure 4:**
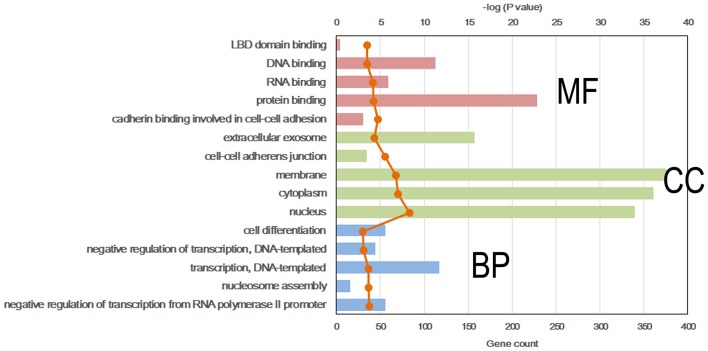
Top 5 significant GO term of differentially methylated genes in each classification. Red, molecular function (MF); Green, cellular component (CC); Blue, biological process (BP).

**Figure 5:**
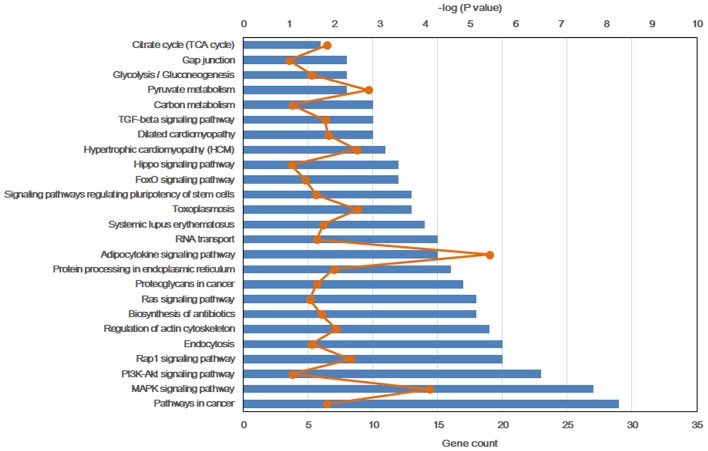
Top 25 significant KEGG pathways of differentially methylated genes.

To further explore the molecular mechanism by which the offspring were exposed to a HF diet in an intrauterine environment, DMR-related genes were analyzed using GO functional analysis and KEGG enrichment analysis. Significantly enriched GO terms of DMR-related genes mainly participating in molecular function are LBD domain binding, DNA binding, RNA binding, protein binding, and cadherin binding involved in cell-cell adhesion (Figure 4, Supplemental Table 1). The top 25 KEGG pathways of DMGs are enriched in the adipocytokine signaling pathway, MAPK signaling pathway, pyruvate metabolism, toxoplasmosis, hypertrophic cardiomyopathy, Rap1 signaling pathway, regulation of actin cytoskeleton, protein processing in endoplasmic reticulum, dilated cardiomyopathy, pathways in cancer, citrate cycle, TGF-beta signaling pathway, systemic lupus erythematosus, biosynthesis of antibiotics, proteoglycans in cancer, RNA transport, signaling pathway regulating pluripotency of stem cells, glycolysis/gluconeogenesis, Ras signaling pathway, FoxO signaling pathway, PI3K-Akt signaling pathway, carbon metabolism, hippo signaling pathway, and gap junction (Figure 5, Supplemental Table 2).

### Overall Differential DNA Methylation of *Irs2* and *Map2k4*

**Figure 6:**
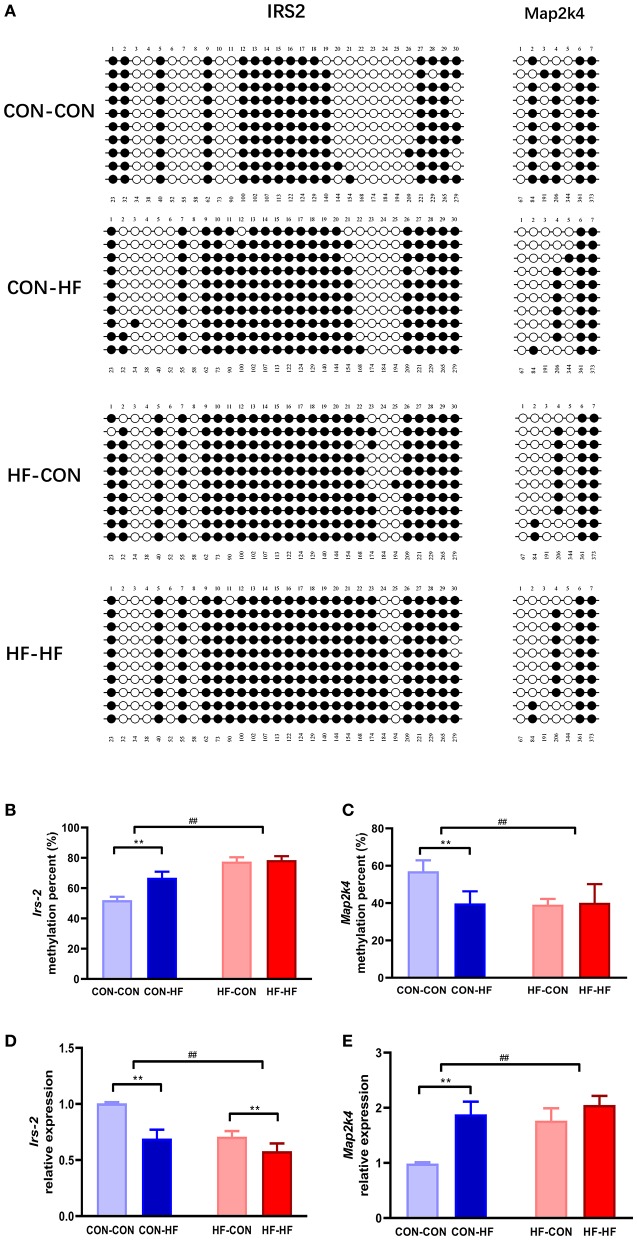
Validation of methylation array using bisulphite sequencing. **(A)** Schematic diagram of bisulphite sequencing results on 30 CpG sites on *Irs2* and 7 CpG on *Map2k4*. Open circles indicate unmethylated CpGs, and closed circles indicate methylated CpGs. Methylation ratio of *Irs2* **(B)** and *Map2k4* **(C)** in different groups. Relative gene expression of *Irs2* **(D)** and *Map2k4* **(E)** in different groups. **P < 0.01 offspring diet effect; ^*##*^*P* < 0.01 maternal diet effect. Values are mean ± SEM (*n* = 10). CON-CON, control diet mother-postweaning control diet; CON-HF, control diet mother-postweaning high-fat diet; HF-CON, high-fat diet mother-postweaning control diet; HF-HF, high-fat diet mother-postweaning high-fat diet.

KEGG pathway analysis revealed that insulin signaling and MAPK may be mainly enriched among the DMGs regulated by pup livers from maternal HF diet-fed mice. To verify the methylation levels of candidate genes revealed by the methylation array, we further studied *Irs2* and *Map2k4*, which are involved in the insulin signaling pathway and the MAPK pathway. As shown in Figures 6A,B, the *Irs2* gene promoter region underwent a series of hypermethylations in all HF-fed groups, and this was more pronounced in pups from HF diet dams (*P* < 0.01). *Map2k4* gene methylation was inhibited in CON-HF mice compared with CON-CON mice (*P* < 0.01, Figures 6A,C). The maternal HF diet had an inhibitory effect on *Map2k4* gene methylation in offspring (*P* < 0.01, Figures 6A,C).

### Gene Expression of *Irs2* and *Map2k4*

qPCR was applied to confirm the abnormal expression of *Irs2* and *Map2k4* in the livers from offspring exposed to a HF diet *in utero* or in adulthood. As illustrated in Figure 6D, the relative expression of *Irs2* mRNA was decreased markedly by both a maternal HF diet and postweaning HF diet (*P* < 0.01). *Map2k4* mRNA expression increased significantly in HF diet-fed mice (*P* < 0.01, Figure 6E). Furthermore, those exposed to maternal HF diet displayed increased *Map2k4* mRNA levels (*P* < 0.01, Figure 6E).

## Discussion

Our results showed that the postweaning HF diet increased body weight in mice. However, the maternal HF diet did not change pup body weight at 8 weeks of age. While some studies reported that HF diet-fed offspring exposed to a maternal HF diet had higher body weight in the adult period (26), other studies did not report any difference (27). This discrepancy may be because of different dietary components, fat sources or the strain and sex of the mice used. Our results revealed that both the maternal HF diet and postweaning HF diet led to glucose intolerance in male mice. Similar observations have been made in a previous study (26, 28–30). A systematic review of animal models found that male offspring exposed to a maternal HF diet independent of maternal obesity, birth weight or postweaning macronutrient intake had glucose intolerance (31). Additionally, a HF diet during fetal life, particularly if combined with the same insult during the suckling period, can induce the type 2 diabetes phenotype (32–34). Maternal obesity interacted with the postweaning HF diet to induce higher levels of glucose intolerance in offspring rodents (35, 36). This programming effect may have sex differences and lead to liver transcriptome changes (37). We also found that fasting insulin levels increased significantly both in mice exposed to the maternal HF diet and postweaning HF diet. In several studies on rats, offspring exposed *in utero* to a HF diet had increased fasting insulin levels (34, 38). In our study, both maternal HF diet and postweaning HF diet caused insulin resistance in pups. The discordance between an increased HOMA index and lack of an increase in plasma insulin levels may be because of the high fasting blood glucose levels in pups exposed to a maternal HF diet. Previous studies have reported that offspring exposed to a maternal HF diet *in utero* and/or postnatally developed manifestations of insulin resistance (29, 39).

A HF diet may affect the epigenetic status through several pathways. On the one hand, a HF diet can provoke inflammation and hormone secretion and alter DNA methylation (40). On the other hand, a HF diet can also act directly on epigenetic modification and methylation pathways. A short-term HF diet in healthy men induces widespread DNA methylation changes in the skeletal muscle. These changes were only partially reversed after 6–8 weeks (41). Roux-en-Y gastric bypass (RYGB)-induced weight loss is associated with the restoration of DNA methylation changes caused by obesity in skeletal muscle (42) and adipose tissue (43, 44). These DNA methylation changes involve pathways that control lipid metabolism and mitochondrial function in skeletal muscle (42), adipogenesis (43), and insulin-mediated glucose (44) uptake in adipose tissue.

A maternal HF diet may also induce DNA methylation changes in several metabolic genes (45) and their expression (37) in male offspring livers. In rats, the hepatic cell cycle inhibitor Cdkn1a was hypomethylated in offspring from HF diet-fed mothers (46). Increased methylation of the leptin promoter and decreased methylation of Ppar-α was also observed in the liver of female offspring from HF diet-fed dams (47). The maternal diet also increased Ppar-γ and liver X receptor α (LXRα) DNA methylation levels in male mouse livers (48). The present study used genomic DNA methylation technologies to examine DNA methylation profiles in mice exposed to a HF diet. First, to address whether maternal HF diet exposure induced methylation changes, we compared HF-CON and CON-CON mice. One thousand 99 DMRs were identified, and gene-associated DMRs clustered mainly in 25 pathways. We then compared DNA methylation in the identified regions among all four groups of mice to uncover the impact of HF diet intake regardless of timing. From these metabolic pathways, we concluded that the HF diet decreases *Map2k4* DNA methylation and increases *Irs2* DNA methylation, as all of the HF-CON, CON-HF, and HF-HF groups generally had lower *Map2k4* DNA methylation and higher *Irs2* DNA methylation than the CON-CON group.

Previous studies revealed that exposure to both undernutrition and overnutrition *in utero* induces gene-specific DNA methylation modification (49–52) in animal models. Several clinical studies revealed that genome-wide DNA methylation significantly changed in offspring born to overweight, obese or malnourished mothers (53–57). These DNA methylation modifications in offspring cord blood involve cardiovascular, inflammatory and apoptosis pathways (55, 57). Moreover, exposure to a maternal HF diet *in utero* might affect glucose and lipid metabolism of female offspring through epigenetic modifications to adiponectin and leptin genes even for multiple continuous generations (58). Exposure to normal diet *in utero* in the subsequent generations after HF diet exposure for three generations did not completely reverse the changes (59). However, maternal short-term transition from a HF diet to a normal diet before and during pregnancy and lactation without weight loss is not beneficial and even aggravated offspring obesity (60).

These observed methylation modifications affected by a maternal HF diet may result from several mechanisms during early life. In the perinatal period, *de novo* methyltransferases, such as Dnm3a and Dnm3b, block *de novo* methylation, which is important in normal development and disease (61). However, during the postweaning period, DNA methyltransferase DNMT1 carried out *de novo* and non-CG methylation (62). A HF diet *in utero* may affect these methyltransferases. Folate- and methyl-deficient diets *in utero* affect methyltransferase expression, including DNMT1 and DNMT3 (63–66). The HF diet affected the expression of DNMTs and their binding to the leptin promoter (67).

The molecular pathway analysis based on the KEGG database revealed that the differentially methylated *Mapk* gene was enriched. In our research, the *Map2k4* gene was hypomethylated in pups exposed to a maternal HF diet. *Map2k4* gene expression increased in the HF-CON, CON-HF, and HF-HF groups compared with expression in the CON-CON group. MAPKs consist of p38 MAPK, JNK, and ERK (68, 69). Activated MAPKs can inhibit expression of multiple targets, including insulin receptor substrate (IRS) proteins (70, 71) and resulting in the inhibition of insulin activity (72, 73).

Our results demonstrated that the *Irs2* methylation level increased in offspring exposed to a HF diet *in utero*. Moreover, *Irs2* gene expression was reduced in the livers of mice exposed to a HF diet as adults and *in utero*. IRS proteins are key components in the insulin signaling pathway (74, 75). Once stimulated by insulin, IRS is phosphorylated and then triggers intracellular signaling through the recruitment of proteins with the Src homology-2 domain, including PI3K, Grb-2, Nck, fyn, and Shp-2, among others (74, 76–78). Murine experiments reveal that targeted depletion of IRS-1 or IRS-2 leads to insulin resistance (79–82). Clinical and animal experiments prove that T2D patients and insulin-resistant rodents have defects in the phosphorylation of IRS proteins *in vivo* and *in vitro* (83, 84). This evidence proves that IRS defects are the molecular basis for insulin resistance. Hence, our results demonstrated that the maternal HF diet activated hepatic *Irs2* methylation and reduced *Irs2* gene expression. These findings suggest that the potent insulin resistance induced by a maternal HF diet may occur via activation of the key insulin signaling pathway molecule IRS, methylation, and reduced *Irs2* expression.

## Conclusions

**Figure 7:**
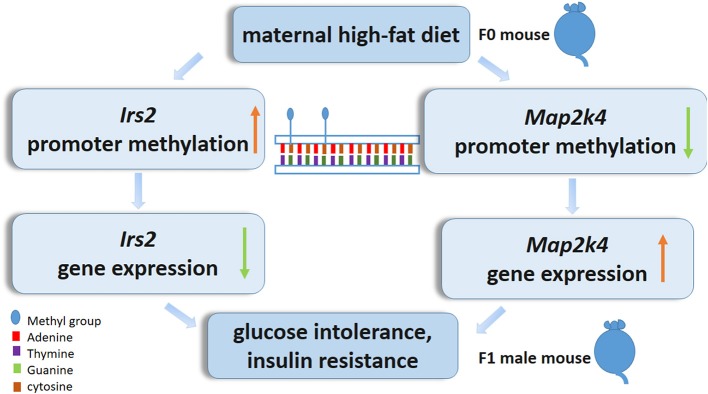
Epigenetic mechanism of high-fat diet *in utero* and adult on offspring. *In utero* expose to high-fat diet modify *Irs2* and *Map2k4* gene methylation and gene expression in offspring, led glucose intolerance, and insulin resistance.

In summary, we characterize the epigenetic alteration profile in the livers of postweaning control diet-fed offspring exposed to a maternal HF diet throughout gestation and lactation. In particular, decreases in *Map2k4* DNA methylation and increases in *Irs2* DNA methylation may play a central role in the livers of pups exposed to a maternal HF diet (Figure 7). More evidence needs to focus on the association of the epigenetic genome-wide status of the liver with the diabetes status in offspring. Furthermore, detailed studies exploring the function of candidate genes in the regulation of the liver are needed and could contribute to the prevention of the morbidity of metabolic-related diseases resulting from maternal HF diets.

## Data Availability Statement

All microarray data have been deposited into the gene expression omnibus (GEO ID: GSE136814) (https://www.ncbi.nlm.nih.gov/geo/query/acc.cgi?acc=GSE136814).

## Ethics Statement

All research procedures involving animals were approved by the Animal Care Committee of Peking Union Medical Hospital (Permit Number: MC-07-6004).

## Author Contributions

XX conceived and designed the experiments. QZ, JZ, TW, and XW performed the experiments. MY, ML, and FP analyzed the data. XX contributed reagents, materials, and analysis tools. QZ wrote the paper.

### Conflict of Interest

The authors declare that the research was conducted in the absence of any commercial or financial relationships that could be construed as a potential conflict of interest.
