# Supplementary material for: A Maternal High-Fat Diet Induces DNA Methylation Changes That Contribute to Glucose Intolerance in Offspring
Source: Front Endocrinol (Lausanne). 2019 Dec 13;10:871. doi: 10.3389/fendo.2019.00871 (PMC6923194; doi:10.3389/fendo.2019.00871)
Supplement: Supplementary file 1 [file Table_1.DOCX]

**Supplemental Table 1 DMG GO analysis**

| Term ID | Term name | Count | PValue | Genes |
| --- | --- | --- | --- | --- |
| GO:0000122 | negative regulation of transcription from RNA polymerase II promoter | 55 | 1.97E-04 | NOG, MEF2A, HFE2, EZH2, PAX4, REST, DAXX, GLI3, TGFB1, CITED2, MEN1, DNAJC17, ZGPAT, EDNRB, KDM5A, TWIST2, ZFP366, DAB2IP, RCOR1, TLE1, RBBP7, PROX1, MSC, KDM2B, VEGFA, TGIF2, RIPPLY2, MED1, EID1, FGFR3, HDGF, HIST2H3C1, TAGLN3, NR2C1, HIC1, EZR, HEXIM1, PTH, OTUD7B, TBX6, SMAD7, WWTR1, SNAI2, PPP1R13L, SNAI3, BRMS1, PDE2A, NEDD4, ETS2, HIST1H3C, PHF21A, PTCH1, HIST1H3E, TBL1X, NCOR2 |
| GO:0006334 | nucleosome assembly | 15 | 2.20E-04 | HIST2H2AA2, HIST1H2BB, BRD2, HIST1H2BG, HIST2H3C1, SART3, NAP1L5, DAXX, HIST2H2AA1, HIST1H2BM, HIST1H4A, HIST1H3A, HIST1H3C, HIST1H3E, HIST1H3G |
| GO:0006351 | transcription, DNA-templated | 117 | 2.28E-04 | AKNA, MEF2A, INO80, HOXD1, REST, CNOT7, CITED1, ZFP580, ZIC3, CITED2, ZGPAT, DDX17, FIZ1, TWIST2, RCOR1, FOXN1, MTA1, PROX1, KDM2B, ZFP280C, ZFP263, TGIF2, AKAP8, NFE2L2, EID1, CNBP, ONECUT1, HDGF, SOX7, MYT1, ZFP768, NR2C1, HEXIM1, SMARCB1, LHX3, HNRNPD, NKX3-1, SLC30A9, HELLS, HIP1, ZBTB46, SMAD7, SMAD5, SAFB2, PAXIP1, ETS2, HOXB5, IKBKG, DDX54, FOXI1, NCOR2, ZFP12, HLF, FOXK1, GPBP1, ELF4, EZH2, ARID4B, PAX4, DAXX, GLI3, TMF1, DNAJC17, MEN1, FUBP1, HLX, SPIB, ALX4, KDM5A, SOX10, SNAPC4, TLE1, TLE2, RBBP7, NLRP3, MSC, PCGF1, CCNL2, SENP2, HOXC10, ITGB1BP1, USP22, MED1, THOC1, ZFP397, PML, TXLNG, HIC1, TSC22D1, TRIM30A, PRDM12, HIC2, POU2F3, SAFB, ZFP524, WAC, LIMD1, TLX2, ZFP213, TBX6, BRD2, BRD3, TRNP1, ZFP703, ILF3, WWTR1, SNAI2, PPP1R13L, POLR3E, SNAI3, BRMS1, SP2, MAPK13, PHF21A, IRF4, TBL1X, KDM4D |
| GO:0045892 | negative regulation of transcription, DNA-templated | 44 | 8.11E-04 | ZFP12, EID1, FOXK1, NFKBIE, EZH2, PML, SOX7, PAX4, REST, DAXX, GLI3, CITED1, TGFB1, NR2C1, CITED2, MEN1, ZGPAT, DAB2, HEXIM1, DNMT3L, A430033K04RIK, NKX3-1, LIMD1, LOXL3, TWIST2, SOX10, DAB2IP, NACC2, RCOR1, ZFP703, TLE1, ILF3, TLE2, RBBP7, PROX1, DUSP5, CD38, BRMS1, CDKN1B, ZFP280C, ZFP263, PTCH1, TBL1X, NCOR2 |
| GO:0030154 | cell differentiation | 55 | 9.78E-04 | NOG, MEF2A, FOXK1, ELF4, PAX4, ZIC3, CITED1, CITED2, NDE1, DAB2, DAB1, HLX, PUM1, PIWIL1, RHOB, PIWIL2, TWIST2, ANAPC2, SOX10, EGFL7, PLD6, SNAPC4, CDHR2, FOXN1, MGP, BOLL, ITGB1BP1, VEGFA, STMN1, GADD45B, TNFAIP2, UNC13A, EID1, CPLX2, FGFR3, ONECUT1, STK11, ADAD1, MYT1, HSPA1L, SMARCB1, LHX3, TDRD1, TEC, HIP1, SMAD5, KDR, TXNDC12, SEMA6B, D1PAS1, JAK2, TLL2, BMP8B, LRP4, UTP14B |
| GO:0005634 | nucleus | 339 | 5.15E-09 | HIST2H2AA2, KIFC1, MEF2A, U2AF2, PNKD, RNF216, REST, SART3, ACBD5, CITED1, CITED2, ISG20, HIST2H2AA1, ZGPAT, DDX17, HIST1H2BM, BRPF1, INTS7, A430033K04RIK, PDHA1, FIZ1, TWIST2, PITX2, POLL, TXNL1, ANAPC2, RCOR1, EXD2, PROX1, DPCD, RAD1, CD38, GLUL, SPAG7, MAPK6, ZFP280C, KRT15, VEGFA, TGIF2, MDH2, NEK7, NEK1, DUSP10, IGF2BP1, UBA5, PPT1, HSPA1A, MYT1, ANGEL1, TAGLN3, ASB15, HNRNPA3, DUSP18, PFN1, TPI1, UPRT, HEXIM1, RIBC2, RAC1, LHX3, BRIX1, DNMT3L, FBXO5, SUPV3L1, PRPF40A, HIP1, RECQL4, DHX9, ZBTB46, NOC4L, SMAD7, NXF7, MAP2K4, SMAD5, DUSP23, NDC80, ACACB, KDR, UPF3A, PAXIP1, ETS2, BTBD10, ZFP341, ACAD11, FOXI1, NCOR2, POP7, UTP14B, ABCF1, HLF, ZMAT4, GAR1, ELF4, EZH2, CWC15, VPS37A, RNF187, NAP1L5, DAXX, GLI3, DNAJC17, TMEM109, RRP1B, HLX, CCNY, TUBB6, USP11, SPIB, CCNO, XLR5B, LDHAL6B, LYN, SNAPC4, TLE6, ZDHHC7, TLE1, TLE2, RAD52, RBBP7, SENP2, DOK1, MGEA5, ESRP1, RIPPLY2, NCAPH2, USP22, GADD45B, CRYBA1, PRPF38B, THOC1, MED1, MIDN, HIST1H2AE, PML, POLA1, TXLNG, HIST2H3C1, AZIN1, TRIM30A, ZFP36L2, WDR18, PLIN2, REXO4, LIMD1, WAC, RBM27, TLX2, ZFP213, ZBP1, TBX6, HIST1H2BB, BRD2, BRD3, HIST1H2BG, ZFP703, BIRC7, ZFP119B, ILF3, SNAI2, CAPN2, WWTR1, POLR3E, MIS12, SNAI3, RASSF5, BRMS1, PDE2A, PPP1R7, RPS6KA1, PTP4A3, MEX3D, PHF21A, MPHOSPH6, TOB2, AKNA, PASK, INO80, RPS27L, HOXD1, UBQLN1, ANKRD6, CNOT7, ZIC3, ZFP580, TGFB1, TOP1, DAB2, ANK1, UBXN2A, TIAM1, ORC6, ACIN1, CUTC, SNRPA1, PHRF1, C2CD2, NUDT4, NUDT3, DTL, FMR1, FOXN1, GM9376, SIX2, MTA1, RSL1D1, RPAIN, KDM2B, LLPH, ZFP263, SNTG1, NFE2L2, AKAP8, DST, TNFAIP1, 1110037F02RIK, EP400, EID1, ELAC1, CNBP, FGFR3, ONECUT1, STK11, HDGF, SOX7, NEDD8, LIN28A, SESN2, ZFP768, NR2C1, POLE4, PPP1R16B, HIST1H4A, POLE3, SMARCB1, OTUD7B, HNRNPD, EIF3K, NKX3-1, NDRG1, RPL7A, STK19, SLC30A9, TRAF4, HELLS, DNAJA2, PARD6A, FKTN, CINP, MPHOSPH10, SYCE1, ARMC1, SAFB2, TRADD, CDKN1B, NEDD4, HOXB5, HIST1H3A, IKBKG, DDX50, CYFIP2, HIST1H3C, HIST1H3E, DDX54, HIST1H3G, TDPOZ2, DDX51, ZFP12, RAI1, FOXK1, GPBP1, AGTPBP1, ARID4B, PRDX5, PAX4, FAM63A, NDUFAF3, TMF1, FUBP1, MEN1, PAK6, CASP3, RANBP3, PIWIL1, RHOB, RANBP1, RANBP2, KDM5A, ALX4, ZFP366, SOX10, NUP88, TMEM38A, PRKCI, NLRP3, MSC, MCM5, PCGF1, CCNL2, RFWD2, CD3EAP, HOXC10, PRKCQ, ZPBP2, ITGB1BP1, CPSF3, ANKRD34B, ZFP397, ADAD1, ADH5, PPM1A, HIC1, KRT9, TSC22D1, PRDM12, HIC2, SAFB, POU2F3, TOR1A, ZFP524, PLCD1, TERF1, NOX4, MSH6, MORN3, ADARB1, NACC2, TRNP1, PPP1R13L, PARK7, CENPI, GSG2, SP2, ANXA11, JAK2, DENND4B, IRF4, C77370, TBL1X, KDM4D |
| GO:0005737 | cytoplasm | 360 | 8.98E-08 | MEF2A, PITPNA, PNKD, PDLIM1, RNF216, AMOTL1, SART3, PRKG1, CITED1, CITED2, ISG20, AQP2, ATCAY, HIST1H2BM, DHX37, IFT20, HOMER3, FIZ1, TWIST2, PITX2, CDCA3, TXNL1, GTPBP1, DAB2IP, EXD2, PROX1, BOLL, PITPNM1, GLUL, MAPK6, VEGFA, TMEM184A, RPS11, EIF2AK3, UNC13A, NEK7, ADSS, TPPP3, 1810043G02RIK, NFKBIE, NEK1, DUSP10, IGF2BP1, UBA5, HSPA1A, RFFL, POMC, MYT1, CAPZB, ASB15, RAB7, HNRNPA3, DUSP18, PFN1, RILPL1, FAM65C, UPRT, HEXIM1, RAC1, ZDHHC9, DNMT3L, FBXO5, SUPV3L1, TDRD1, PRPF40A, HIP1, RECQL4, DHX9, NXF7, SMAD7, MAP2K4, SMAD5, FBXO2, DUSP23, EHBP1, NDFIP2, EPRS, MAGEB4, TNNI3, S100A14, E130308A19RIK, NCKAP1, KDR, UPF3A, CEL, ETS2, BTBD10, RHEB, POP7, ABCF1, LIMA1, GFAP, NAAA, UBE2G1, EZH2, VPS37A, RNF187, TTC28, DAXX, GLI3, DNAJC17, ST6GALNAC6, NOD2, SLMAP, TUBB6, USP11, FBXL15, CCNO, FBXL16, GOLGA4, ATP5H, PARVG, ARHGEF3, LYN, ASTL, FBXL20, ZDHHC5, TLE6, FBLIM1, TLE1, ARRDC3, SENP2, DOK1, PJA1, MGEA5, PLA2G6, KSR1, GADD45B, RAB11FIP1, CRYBA1, THOC1, ATG10, PML, POLA1, TXLNG, AZIN1, FKBP1A, PLCL1, TRIM30A, ZFP36L2, GPHN, WDR18, EZR, PLIN2, NCAPG, MAN1A, EXOC4, LIMD1, HSF2BP, RBM27, TLX2, RASA2, PLEC, ZBP1, AATK, HIST1H2BB, BRD2, HIST1H2BG, SPSB2, ZFP703, BIRC7, RTN4R, ILF3, SNAI2, CAPN2, WWTR1, AGTRAP, PCK1, FEM1C, RASSF5, TUBA8, BRMS1, PDE2A, RPS6KA1, PTP4A3, MEX3D, CHN2, BAMBI, MPHOSPH6, HPGD, PAICS, TOB2, GMPR2, SRCIN1, CAPZA1, PASK, SLC7A8, IL15, UBQLN1, ANKRD6, CNOT7, ZIC3, TGFB1, TOP1, GSTM3, DAB2, PRKAR2A, ANK1, WNT3, TIAM1, SLC2A1, DNAJC8, PUM1, AKR7A5, ACIN1, CUTC, IRS2, DARS, NUDT4, BST2, NUDT3, DTL, FMR1, SIX2, MTA1, NTSR1, TACC3, ISCU, ARPC1B, TRIM36, RPAIN, RASGRF2, PAM16, ZFP263, SNTG1, MVK, STMN1, AKAP8, NFE2L2, ERC1, DST, LRRK1, TNFAIP1, SEPT9, EID1, ELAC1, FGFR4, CNBP, FGFR3, TMEM214, STK11, HDGF, MAP4K1, SOX7, LIN28A, SESN2, ITGB1, AZI2, EIF3B, FBXW5, BLOC1S3, OTUD7B, DHODH, HNRNPD, EIF3K, EIF3I, NDRG1, RPL7A, SLC30A9, STK38L, TRAF4, FGD3, TEC, PARD6A, GABARAPL1, CUBN, WDR7, MMADHC, BFSP1, IREB2, EVL, KCTD2, SAFB2, TRADD, CDKN1B, NEDD4, PRKAR1B, IKBKG, 4930579G24RIK, CYFIP2, CARS2, PARD6G, MYO19, DNAJB4, TDPOZ2, PDZD4, RAI1, AGTPBP1, LRRC8C, MRVI1, PRDX5, DSTN, TMF1, MEN1, FRMD3, PAK6, CASP3, NDE1, ZFYVE19, DDX60, CTU2, RANBP3, PIWIL1, PIWIL2, RANBP1, KDM5A, TUBA1B, SOX10, ATPAF2, NOL3, PFKL, PRKCI, TBCE, NLRP3, RFWD2, CD3EAP, TMEM67, PRKCQ, ITGB1BP1, ZFYVE28, DDT, AAMP, CLDN1, LCE3F, CPLX2, MMGT1, ANKRD34B, RNH1, ADH5, PPM1A, CDH1, TKTL2, KIF9, TPM4, HIC1, TSC22D1, RASGRP3, POU2F3, TOR1A, GGPS1, PLCD1, VPS35, SERPINB9E, TERF1, MSH6, ADARB1, TMBIM6, DTX3, KY, GDPD5, PPP1R13L, PARK7, CENPI, GSG2, CCDC113, ANXA11, JAK2, IRF4, ARAP3, ARAP1 |
| GO:0016020 | membrane | 375 | 1.67E-07 | KIFC1, VTCN1, ADCY8, OSMR, PNKD, PRKG2, PRKG1, ACBD5, SLC35A3, AQP2, HMHA1, RTBDN, ERO1LB, DDX17, MALL, KCNK9, HOMER3, LPCAT2B, PI16, GTPBP1, F11R, CRTAM, OSBP2, DAB2IP, PDPN, PLD6, CDHR1, CDHR2, LIFR, UBE2J2, CD38, PITPNM1, TMEM136, VSIG1, LHFPL4, LCTL, VEGFA, TMEM184A, MST1R, RPS11, EIF2AK3, MDH2, UNC13A, ITGA2B, CLCN3, CRELD1, RER1, SFXN1, BDKRB1, PPT1, RFFL, GPR143, CAPZB, SFXN5, RAB7, DUSP18, TMEM129, PFN1, TRAM2, RILPL1, RAC1, ZDHHC9, PRPF40A, HIP1, RECQL4, MGAT4B, DHX9, PLB1, NOC4L, FBXO2, RHBDF1, EHBP1, NDFIP2, EPRS, NDC80, ACACB, NCKAP1, KDR, SEMA6B, WSCD1, GOLGA7B, ATP2A3, OLFR474, RHEB, GHSR, CLCN7, NCOR2, ABCF1, GFAP, LEPROTL1, CACHD1, CYP2J6, VPS37A, CD151, MMP25, VEPH1, EDNRB, ST6GALNAC6, NOD2, TMEM109, ADCK2, SLMAP, ATG2A, CCNY, GPX8, GOLGA4, ATP5H, COX15, OLFR18, PARVG, PIK3C2G, JKAMP, LYN, CMKLR1, ZDHHC5, PGAP1, ZDHHC7, SDK2, CACNG5, ARRDC3, ST6GALNAC2, SENP2, MGEA5, PLA2G6, SUSD3, NCAPH2, KSR1, RAB11FIP1, STEAP1, MED1, RAB3A, SLC39A10, GPR63, RPL27A, PML, TXLNG, HIST2H3C1, FKBP1A, MIP, BEST3, GPHN, EZR, PLIN2, NCAPG, IL10RA, MAN1A, COL6A2, ETFDH, EXOC4, FUT4, PCSK7, GLIPR1L2, ACSL3, GALNT14, RASA2, AATK, PIRT, A4GALT, RTN4R, TMEM5, ILF3, CAPN2, AGTRAP, SDHA, CD19, PDE2A, PTP4A3, LAYN, NPHS2, LRRN1, CHN2, GFRA1, PTCH1, PTCH2, BAMBI, PAICS, NCLN, LRP4, AKNA, KCNC4, HFE2, SRCIN1, CTPS, CAPZA1, SLC7A8, JAG1, CNOT7, UBQLN1, C2CD2L, DAB2, PRKAR2A, ANK1, DAB1, CISD1, ST3GAL4, TIAM1, PGRMC1, PGRMC2, SLC2A1, SLC25A29, ORC6, PDGFD, DNAJC4, RNF149, LEPROT, PQLC2, DSCAM, PHRF1, KCND3, TMEM204, DARS, BST2, DTL, ERP29, FMR1, NTSR1, SLC25A30, RSL1D1, RASGRF2, SLC25A34, SLC25A36, VAMP4, STMN1, SLC25A39, RNF26, SLC38A1, AKAP8, ERC1, DST, STX5A, DERL2, FGFR4, FGFR3, TMEM214, PANX1, STK11, ARFRP1, PAQR6, TMEM218, MAP4K1, NINJ1, OPN1SW, FXYD5, ITGB1, PPP1R16B, HIST1H4A, DHODH, EIF3K, ARL6IP6, NDRG1, RPL7A, SLC30A9, STK38L, TRAF4, LRFN3, UPK2, TEC, OTOP1, DNAJA2, PARD6A, GABARAPL1, FKTN, RAB8B, CUBN, GABRA4, BFSP1, DOCK9, RHD, EVL, CACNA2D3, CACNA2D2, ABCB6, SLC6A19, ST7L, DDX55, LOXHD1, NEDD4, GRINA, PRKAR1B, HIST1H3A, NDUFV2, DDX50, CYFIP2, HIST1H3C, CACNA1E, SCN8A, PARD6G, MYO19, HIST1H3E, DNAJB4, DDX54, HIST1H3G, MS4A13, TMEM88, SLC5A11, DDX51, SLC45A3, SLC5A1, LRRC8C, LEPR, MRVI1, HEXB, PCDHGA7, LSS, SDC4, NDUFAF3, TMF1, FRMD3, TAAR6, NDE1, RHOB, RANBP2, LOXL3, DENND5B, EBAG9, PIGA, NOL3, MAN1A2, PFKL, PIGW, TMEM38A, PRKCI, HEPHL1, FSHR, MCM5, TMEM67, FAM134B, PRKCQ, LARP4, MANEAL, DGAT2, PSD, SLC35E3, CHRM1, ITGB1BP1, ZFYVE28, SLC35E1, CLDN1, PPM1L, SNX13, CD101, MMGT1, LMF2, APH1C, PPM1A, CDH1, PCDHGC4, SEC63, TPM4, KRT9, SNN, TMEM37, TAP2, TSPAN32, TOR1A, PLCD1, VPS35, TMEM45A, NDUFA4, NOX4, NDUFA5, 2410015M20RIK, TMBIM6, GDPD5, CYP20A1, TSPAN18, PARK7, RDH11, SLC35C1, ANXA11, JAK2, IRF4, ARAP3, ARAP1, SCN4A |
| GO:0005913 | cell-cell adherens junction | 34 | 2.91E-06 | LIMA1, CAPZA1, HIST2H3C1, CDH1, HSPA1A, PDLIM1, ITGB1, CAPZB, PAK6, PFN1, EZR, RANBP1, NDRG1, RPL7A, DLG5, PLEC, COBLL1, F11R, CRTAM, DAB2IP, LYN, SMAD7, DOCK9, PPP1R13L, PARK7, RSL1D1, HIST1H3A, HIST1H3C, HIST1H3E, ERC1, HIST1H3G, STX5A, PAICS, SEPT9 |
| GO:0070062 | extracellular exosome | 157 | 4.51E-05 | HIST2H2AA2, LTBP2, PITPNA, CAPZA1, SLC7A8, C1QC, HIST2H2AA1, AQP2, APOA4, HIST1H2BM, PRKAR2A, DAB2, CISD1, ST3GAL4, IFT20, PGRMC1, SLC2A1, RNF149, AKR7A5, PDGFD, PI16, MB, TXNL1, F11R, DAB2IP, DARS, BST2, NUDT3, CRTAC1, ERP29, CDHR2, LIFR, MGP, ARPC1B, CD38, GLUL, TRIM36, KRT15, MVK, STMN1, RPS11, SLC38A1, MDH2, ITGA2B, ADSS, TPPP3, STK11, NEDD8, PPT1, CAPZB, ITGB1, RAB7, PFN1, TPI1, EIF3B, HIST1H4A, RAC1, IDH2, EIF3K, HNRNPD, NDRG1, EIF3I, RPL7A, ANGPTL2, UPK2, DNAJA2, RAB8B, CUBN, FBXO2, DUSP23, S100A14, ABCB6, SLC6A19, ECM1, NCKAP1, SAFB2, CEL, NEDD4, HIST1H3A, ARMC9, CYFIP2, ALDH2, HIST1H3C, RHEB, HIST1H3E, DNAJB4, HIST1H3G, NAAA, UBE2G1, SLC5A1, HEXB, CYP2J6, PRDX5, FAM63A, SDC4, DSTN, TMEM109, CCNY, TUBB6, RHOB, ATP5H, TUBA1B, GOLGA4, PPP2R1B, PFKL, MAN1A2, LYN, TMEM38A, PRKCI, ITGB1BP1, DDT, PPM1L, CTSF, CD101, HIST1H2AE, RNH1, ADH5, HIST2H3C1, CDH1, FKBP1A, CPN2, TPM4, KRT9, TMED7, EZR, NHLRC3, TOR1A, MAN1A, COL6A2, VPS35, PLCD1, TSTA3, PLEC, ETFA, NDUFA4, COBLL1, GSTA2, A4GALT, 0610037L13RIK, HIST1H2BG, RNASE6, HSPG2, RTN4R, NID2, CAPN2, PARK7, PCK1, KRT75, CD19, PPP1R7, FBLN2, NPHS2, ANXA11, GFRA1, HPGD, PAICS, TRMT112 |
| GO:0098641 | cadherin binding involved in cell-cell adhesion | 30 | 1.84E-05 | LIMA1, CAPZA1, HIST2H3C1, CDH1, HSPA1A, PDLIM1, ITGB1, CAPZB, PAK6, PFN1, EZR, RANBP1, NDRG1, RPL7A, PLEC, COBLL1, F11R, DAB2IP, DOCK9, PPP1R13L, PARK7, RSL1D1, HIST1H3A, HIST1H3C, HIST1H3E, ERC1, HIST1H3G, STX5A, PAICS, SEPT9 |
| GO:0005515 | protein binding | 228 | 5.81E-05 | KIFC1, MEF2A, PNKD, U2AF2, LDHD, PDLIM1, PRKG2, REST, AMOTL1, PRKG1, CITED1, CITED2, IFT20, HOMER3, FAU, FIZ1, TWIST2, PITX2, F11R, DAB2IP, PLD6, CRTAC1, RCOR1, CDHR1, LIFR, PROX1, GLUL, MAPK6, VEGFA, EIF2AK3, UNC13A, PPT1, HSPA1A, MYT1, CAPZB, RAB7, PFN1, TRAM2, RAC1, DNMT3L, LHX3, FBXO5, TDRD1, HIP1, PRPF40A, DHX9, SMAD7, CRIP2, FBXO2, SMAD5, MAP2K4, EHBP1, NDC80, KDR, NCKAP1, LAMA1, PAXIP1, ATP2A3, BTBD10, TRAFD1, NCOR2, LIMA1, GFAP, GAR1, EZH2, UVRAG, DAXX, GLI3, FBXL19, EDNRB, TMEM109, NOD2, RRP1B, ASTL, LYN, ZDHHC5, TLE6, TLE1, RBBP7, PJA1, KSR1, FKBP10, GADD45B, MED1, THOC1, RAB3A, ATG10, USP5, PML, TXLNG, FKBP1A, MIP, GPHN, EZR, PLIN2, SOS2, EXOC4, LIMD1, TLX2, AATK, ETFA, PLEC, SPSB2, RTN4R, HSPG2, NID2, SNAI2, WWTR1, RASSF5, BRMS1, FBLN2, NPHS2, PTCH1, LRP4, SRCIN1, CAPZA1, JAG1, CNOT7, ANKRD6, ZIC3, TGFB1, PRKAR2A, DAB2, DAB1, WNT3, TIAM1, PGRMC2, SLC2A1, RNF149, LEPROT, SPATA22, DSCAM, KCND3, IRS2, TMEM204, FMR1, MTA1, SIX2, FGF22, TACC3, RSL1D1, LLPH, STMN1, NFE2L2, AKAP8, ERC1, DST, LRRK1, EP400, EID1, FGFR4, FGFR3, STK11, HDGF, MAP4K1, NEDD8, SESN2, ITGB1, NR2C1, FBXW5, POLE3, SMARCB1, BLOC1S3, OTUD7B, NKX3-1, ARL6IP6, RPL7A, TEC, PARD6A, GABARAPL1, RAB8B, CUBN, BFSP1, EVL, SYCE1, SLC6A19, TRADD, PPIF, CDKN1B, NEDD4, IKBKG, CYFIP2, SCN8A, FOXK1, GPBP1, AGTPBP1, LEPR, MRVI1, TMF1, ACOT9, MEN1, FUBP1, NDE1, DDX60, PIWIL1, PIWIL2, RHOB, DLG5, RANBP2, KDM5A, ALX4, TUBA1B, SOX10, NOL3, PRKCI, NLRP3, CCNL2, RFWD2, CD3EAP, PRKCQ, PPM1J, ITGB1BP1, CLDN1, PPM1L, CDH1, SAFB, TAP2, TOR1A, VPS35, PLCD1, TERF1, TRNP1, DTX3, PPP1R13L, PARK7, JAK2, IRF4, TBL1X |
| GO:0003723 | RNA binding | 59 | 7.54E-05 | GAR1, ZMAT4, U2AF2, TRMT2A, EZH2, CWC15, CNOT7, SART3, ISG20, FUBP1, DNAJC17, DDX17, DDX60, PUM1, PIWIL1, PIWIL2, RANBP2, SNRPA1, NUDT4, FMR1, EIF1A, RBBP7, BOLL, RSL1D1, LARP4, KDM2B, ESRP1, RPS11, CPSF3, THOC1, ADAD1, IGF2BP1, LIN28A, HNRNPA3, ZFP36L2, EIF3B, SAFB, BRIX1, HNRNPD, EIF3K, RPL7A, RBM27, PRPF40A, ZBP1, DHX9, ADARB1, NXF7, IREB2, EPRS, ILF3, PARK7, SAFB2, DDX55, D1PAS1, DDX50, MEX3D, DDX54, MPHOSPH6, DDX51 |
| GO:0003677 | DNA binding | 113 | 3.20E-04 | AKNA, HIST2H2AA2, MEF2A, INO80, HOXD1, REST, ZFP580, ZIC3, HIST2H2AA1, TOP1, ZGPAT, HIST1H2BM, ORC6, TWIST2, PITX2, POLL, RCOR1, ANKRD33B, FOXN1, MTA1, SIX2, PROX1, KDM2B, ZFP280C, ZFP263, TGIF2, AKAP8, NFE2L2, EP400, CNBP, ONECUT1, HDGF, SOX7, MYT1, LIN28A, ZFP768, NR2C1, POLE4, HIST1H4A, POLE3, SMARCB1, LHX3, OTUD7B, HNRNPD, NKX3-1, SUPV3L1, RECQL4, DHX9, SMAD7, SMAD5, E130308A19RIK, SAFB2, ETS2, HOXB5, D1PAS1, HIST1H3C, HIST1H3E, ZFP341, FOXI1, NCOR2, HLF, FOXK1, GPBP1, ZMAT4, ELF4, ARID4B, PAX4, GLI3, TMF1, MEN1, FUBP1, FBXL19, HLX, SPIB, ALX4, KDM5A, SOX10, SNAPC4, RAD52, MSC, MCM5, HOXC10, MED1, THOC1, HIST1H2AE, POLA1, PML, HIST2H3C1, HIC1, TRIM30A, PRDM12, ZFP36L2, HIC2, POU2F3, SAFB, SOS2, ZFP524, TLX2, TERF1, ZBP1, TBX6, MSH6, HIST1H2BB, TRNP1, HIST1H2BG, ILF3, SNAI2, GSG2, SNAI3, SP2, PHF21A, IRF4, TBL1X |
| GO:0050693 | LBD domain binding | 4 | 3.34E-04 | PROX1, CITED1, CITED2, MED1 |

**Supplemental Table 2 DMG KEGG analysis**

| Term No | Term name | Gene Count | P Value | Genes |
| --- | --- | --- | --- | --- |
| mmu04920 | Adipocytokine signaling pathway | 15 | 3.76E-06 | PRKAG3, IRS2, STK11, NFKBIE, LEPR, PRKAG2, ACACB, POMC, TRADD, PCK1, PRKCQ, SLC2A1, IKBKG, JAK2, ACSL3 |
| mmu04010 | MAPK signaling pathway | 27 | 8.01E-05 | FGFR4, FGFR3, PPM1A, DUSP10, MAP4K1, HSPA1A, DAXX, TGFB1, HSPA1L, CASP3, RASGRP3, SOS2, RAC1, RASA2, MAP2K4, CACNG5, FGF22, CACNA2D3, CACNA2D2, DUSP5, RPS6KA1, RASGRF2, MAPK13, IKBKG, CACNA1E, STMN1, GADD45B |
| mmu00620 | Pyruvate metabolism | 8 | 0.001743 | LDHAL6B, LDHD, ALDH2, ACACB, DLAT, PDHA1, MDH2, PCK1 |
| mmu05145 | Toxoplasmosis | 13 | 0.003005 | PPIF, HSPA1L, LAMA1, CASP3, H2-OA, MAPK13, IL10RA, IKBKG, BIRC7, JAK2, HSPA1A, ITGB1, TGFB1 |
| mmu05410 | Hypertrophic cardiomyopathy (HCM) | 11 | 0.003118 | PRKAG3, MYL3, PRKAG2, CACNG5, CACNA2D3, TNNI3, ITGB1, CACNA2D2, TPM4, TGFB1, ITGA2B |
| mmu04015 | Rap1 signaling pathway | 20 | 0.004337 | PARD6A, FGFR4, FGFR3, ADCY8, PRKCI, CDH1, FGF22, ITGB1, KDR, PFN1, RASSF5, RASGRP3, MAPK13, TIAM1, VEGFA, RAC1, PARD6G, PDGFD, ARAP3, ITGA2B |
| mmu04810 | Regulation of actin cytoskeleton | 19 | 0.008928 | FGFR4, FGFR3, FGF22, BDKRB1, ITGB1, NCKAP1, MYL9, PAK6, ARPC1B, PFN1, EZR, TIAM1, CHRM1, SOS2, RAC1, CYFIP2, PDGFD, FGD3, ITGA2B |
| mmu04141 | Protein processing in endoplasmic reticulum | 16 | 0.010145 | DERL2, MAN1A2, UBE2G1, ERP29, FBXO2, HSPA1A, UBE2J2, UBQLN1, CAPN2, SEC63, HSPA1L, ERO1LB, MAN1A, NFE2L2, EIF2AK3, DNAJA2 |
| mmu05414 | Dilated cardiomyopathy | 10 | 0.013405 | ADCY8, MYL3, CACNG5, CACNA2D3, TNNI3, ITGB1, CACNA2D2, TPM4, TGFB1, ITGA2B |
| mmu05200 | Pathways in cancer | 29 | 0.014088 | FGFR3, ADCY8, PML, CDH1, BDKRB1, GLI3, ITGB1, TGFB1, EDNRB, CASP3, WNT3, RASGRP3, RAC1, SOS2, SLC2A1, NKX3-1, TRAF4, MSH6, WNT10A, BIRC7, FGF22, LAMA1, RASSF5, CDKN1B, IKBKG, VEGFA, PTCH1, PTCH2, ITGA2B |
| mmu00020 | Citrate cycle (TCA cycle) | 6 | 0.014345 | SDHA, IDH2, DLAT, PDHA1, MDH2, PCK1 |
| mmu04350 | TGF-beta signaling pathway | 10 | 0.015507 | PPP2R1B, NOG, INHBE, SMAD7, SMAD5, TGIF2, BAMBI, TGFB1, BMP8B, PITX2 |
| mmu05322 | Systemic lupus erythematosus | 14 | 0.017307 | HIST2H2AA2, HIST1H2BB, HIST1H2BG, HIST1H2AE, HIST2H3C1, C1QC, HIST2H2AA1, HIST1H2BM, HIST1H4A, H2-OA, HIST1H3A, HIST1H3C, HIST1H3E, HIST1H3G |
| mmu01130 | Biosynthesis of antibiotics | 18 | 0.019159 | LDHAL6B, PFKL, ADH5, LSS, TKTL2, DLAT, PPAT, PCK1, SDHA, TPI1, HMGCS2, ALDH2, GGPS1, IDH2, MVK, PDHA1, PAICS, MDH2 |
| mmu05205 | Proteoglycans in cancer | 17 | 0.024118 | WNT10A, HSPG2, SDC4, ITGB1, TGFB1, KDR, CASP3, WNT3, EZR, ANK1, TIAM1, MAPK13, VEGFA, RAC1, SOS2, PTCH1, TWIST2 |
| mmu03013 | RNA transport | 15 | 0.024186 | ELAC1, NUP88, NXF7, FMR1, EIF1A, TACC3, UPF3A, SENP2, EIF3B, CYFIP2, EIF3I, RANBP2, ACIN1, POP7, THOC1 |
| mmu04550 | Signaling pathways regulating pluripotency of stem cells | 13 | 0.024437 | WNT10A, FGFR4, WNT3, FGFR3, ONECUT1, INHBE, MAPK13, SMAD5, LIFR, JAK2, REST, ZIC3, PCGF1 |
| mmu00010 | Glycolysis / Gluconeogenesis | 8 | 0.030809 | TPI1, PFKL, LDHAL6B, ALDH2, ADH5, DLAT, PDHA1, PCK1 |
| mmu04144 | Endocytosis | 20 | 0.031024 | PARD6A, FGFR4, FGFR3, CAPZA1, PRKCI, PML, VPS37A, HSPA1A, CAPZB, RAB7, HSPA1L, ARPC1B, DAB2, PSD, NEDD4, VPS35, PARD6G, ARAP3, RAB11FIP1, ARAP1 |
| mmu04014 | Ras signaling pathway | 18 | 0.034101 | FGFR4, FGFR3, FGF22, KDR, PAK6, RASSF5, RASGRF2, RASGRP3, TIAM1, ETS2, VEGFA, IKBKG, SOS2, RAC1, PLA2G6, PDGFD, KSR1, RASA2 |
| mmu04068 | FoxO signaling pathway | 12 | 0.0436 | PRKAG3, IRS2, GABARAPL1, CDKN1B, HOMER3, STK11, MAPK13, SOS2, PRKAG2, GADD45B, TGFB1, PCK1 |
| mmu04151 | PI3K-Akt signaling pathway | 23 | 0.083719 | CSF3, PPP2R1B, FGFR4, FGFR3, OSMR, STK11, FGF22, ITGB1, PCK1, KDR, LAMA1, CD19, CDKN1B, CHRM1, VEGFA, IKBKG, SOS2, RAC1, COL6A2, RHEB, JAK2, PDGFD, ITGA2B |
| mmu01200 | Carbon metabolism | 10 | 0.084825 | SDHA, TPI1, PFKL, MCEE, ADH5, IDH2, TKTL2, DLAT, PDHA1, MDH2 |
| mmu04390 | Hippo signaling pathway | 12 | 0.086612 | PARD6A, PPP2R1B, WNT10A, WNT3, PRKCI, CDH1, LIMD1, PARD6G, WWTR1, SNAI2, TGFB1, BMP8B |
| mmu04540 | Gap junction | 8 | 0.099142 | TUBA8, ADCY8, SOS2, TUBB6, PDGFD, PRKG2, PRKG1, TUBA1B |
